# Multi-Step Unfolding and Rearrangement of α-Lactalbumin by SDS Revealed by Stopped-Flow SAXS

**DOI:** 10.3389/fmolb.2020.00125

**Published:** 2020-07-10

**Authors:** Grethe Vestergaard Jensen, Jannik Nedergaard Pedersen, Daniel E. Otzen, Jan Skov Pedersen

**Affiliations:** ^1^Interdisciplinary Nanoscience Center (iNANO), Aarhus University, Aarhus, Denmark; ^2^Materials Division, Danish Technological Institute, Taastrup, Denmark; ^3^Department of Molecular Biology and Genetics, Aarhus University, Aarhus, Denmark; ^4^Department of Chemistry, Aarhus University, Aarhus, Denmark

**Keywords:** supramolecular complex structures, protein-SDS interactions, stopped-flow kinetics, synchrotron SAXS, circular dichroism, fluorescence, **α**-lactalbumin, unfolding mechanisms

## Abstract

Interactions between proteins and surfactants are both of fundamental interest and relevant for applications in food, cosmetics and detergency. The anionic surfactant sodium dodecyl sulfate (SDS) denatures essentially all proteins. Denaturation typically involves a number of distinct steps where growing numbers of SDS molecules bind to the protein, as seen in multidisciplinary approaches combining several complementary techniques. We adopt this approach to study the SDS-induced unfolding of Ca^2+^-depleted α-lactalbumin (aLA), a protein particularly sensitive toward denaturation by surfactants. By combining stopped-flow mixing of protein and surfactant solutions with stopped-flow synchrotron small-angle X-ray scattering (SAXS), circular dichroism (CD) and Trp fluorescence, together with information from previous calorimetric studies, we construct a detailed picture of the unfolding process at the level of both protein and surfactant. A protein-surfactant complex is formed within the dead time of mixing (2.5 ms). Initially a cluster of SDS molecules binds asymmetrically, i.e., to one side of the protein, after which aLA redistributes around the SDS cluster. This occurs in two kinetic steps where the complex grows in number of both SDS and protein molecules, concomitant with protein unfolding. During these steps, the core-shell complex undergoes changes in shell thickness as well as core shape and radius. The entire process is very sensitive to SDS concentration and completes within 10 s at an SDS:aLA ratio of 9, decreasing to 0.2 s at 60 SDS:aLA. The number of aLA molecules per SDS complex drops from 1.9 to 1.0 over this range of ratios. While both CD and Trp kinetics reveal a fast and a slow conformational transition, only the slow transition is observed by SAXS, indicating that the protein-SDS complex (which is monitored by SAXS) adjusts to the presence of the unfolded protein. We attribute the rapid unfolding of aLA to its predominantly α-helical structure, which persists in SDS (albeit as isolated helices), enabling aLA to unfold without undergoing major secondary structural changes unlike β-sheet rich proteins. Nevertheless, the overall unfolding steps are broadly similar to those of the more β-rich protein β-lactoglobulin, suggesting that this unfolding model is representative of the general process of SDS-unfolding of proteins.

## Significance Statement

Surface-active agents (surfactants) come into contact with proteins in food, cosmetics and detergency. Charged surfactants interact strongly with proteins; the anionic surfactant sodium dodecyl sulfate (SDS) denatures essentially all proteins. This process is of fundamental interest to our understanding of protein conformational stability and dynamics. Previous studies have mainly focused on the changes to the protein component alone. To construct a detailed picture of the unfolding process at the level of both protein and surfactant, we here combine conventional spectroscopy, which monitors protein unfolding, with small-angle X-ray scattering, which reports on the size and shape of protein-surfactant complexes. We use these approaches together with rapid mixing techniques to monitor the SDS-induced unfolding of α-lactalbumin (aLA), which is sensitive toward surfactants. Within the deadtime of mixing, a cluster of SDS molecules binds to one side of the protein, after which aLA redistributes around the SDS as the complex grows in number of both SDS and protein molecules, accompanied by protein unfolding and changes in shell thickness, core shape and radius. The overall unfolding steps are broadly similar to those of the more β-rich protein β-lactoglobulin, suggesting that this unfolding model is representative of the general process of SDS-unfolding of proteins.

## Introduction

The anionic surfactant sodium dodecyl sulfate (SDS) is a very potent general protein denaturant, thanks to its high anionic charge in the micellar state and strong hydrophobic and electrostatic interactions with proteins (Tanford, 1980; Otzen, 2011). This generic unfolding ability is exploited in the universally used separation technique SDS-PAGE, where unfolded SDS-protein complexes on a gel are separated by an electric field based on their size/mass (Laemmli, 1970). A range of physical characterization techniques has been applied to further understand the mechanism of protein unfolding by SDS, and the basis for SDS' superior denaturing abilities. Spectroscopic techniques such as circular dichroism (CD) and Trp fluorescence, which monitor changes to the secondary and tertiary structure of the protein, respectively, reveal that the protein unfolds stepwise with increasing surfactant concentration. Isothermal titration calorimetry (ITC) reports on the exothermic or endothermic nature of these binding steps and, importantly, provides the number of surfactants bound to the protein at each step, when the protein concentration is varied. Finally, small-angle X-ray scattering (SAXS) provides information on the overall size and shape of the protein-surfactant complex, including crucial information such as the distribution of protein around micelles and *vice versa*. Generally speaking, interactions start well-below the critical micelle concentration (cmc), with the binding of a few individual SDS molecules, often to positively charged patches on the proteins [although SDS can bind quite well to proteins completely devoid of charged side chains (Højgaard et al., 2018)]. This binding step needs not involve loss of native structure if individual SDS molecules bind to sites only available in the native state. However, further addition of SDS leads to unfolding, generally in two steps. The first step is associated with loss of tertiary structure, and rearrangement or loss of secondary protein structure, providing more binding sites for the SDS molecules. The second step is largely observed as a change in the Trp environment. SAXS has shown that the second step in several cases corresponds to binding of entire SDS micelles with the protein wrapped around it to decorate the surface of the micelle.

In a recent study, the unfolding and refolding kinetics of β-lactoglobulin (bLG) was followed by stopped-flow synchrotron SAXS, Trp fluorescence, and CD after mixing of protein and surfactant solutions (Pedersen et al., 2019). bLG-SDS complexes are formed within a few milliseconds, and the model which fits the data best has the protein asymmetrically distributed around the SDS micelle in these initial complexes. The complexes are arranged in aggregates, which disintegrate before the protein redistributes on the micelle surface to form the final complex, in which the protein is wrapped symmetrically around the micelle. In addition to these unfolding studies, SDS-unfolded bLG was refolding by addition of the non-ionic surfactant octaethyleneglycol dodecyl ether (C_12_E_8_), which is known to extract SDS from the protein, allowing it to refold (Kaspersen et al., 2017). Stopped-flow studies of this process (Pedersen et al., 2019) showed that a fraction of SDS depleted random-coil proteins are formed already in the deadtime of the mixing and that these proteins refold first. Another fraction of SDS-protein complexes persists for longer times. From these, the SDS molecules have to be removed by transfer to mixed SDS-C_12_E_8_ micelles before the protein can refold.

Here, we investigate the unfolding kinetics of the Ca^2+^-depleted α-lactalbumin (aLA) protein by SDS. aLA and bLG are both globular proteins, which together make up 90% of the protein fraction in whey. aLA is more sensitive toward SDS unfolding than bLG, and it cannot be refolded upon addition of C_12_E_8_; in fact, even non-ionic surfactants like C_12_E_8_ and alkyl maltosides lead to partial unfolding of the protein (Otzen et al., 2009). Whereas, bLG is largely composed of β-strands, aLA is largely α-helical. Although aLA is stabilized by four disulfide bonds and Ca^2+^, both the apo- and holo-form are easily unfolded at low SDS concentrations (Otzen et al., 2009). Unlike β-strands, isolated α-helices (which arise during the unfolding process) are stable in the presence of SDS micelles, and this likely contributes to the increased susceptibility of aLA to SDS. Over the last few decades there has been particular interest in the interactions of aLA with amphiphilic molecules, since aLA is known to form complexes with *cis*-unsaturated fatty acids such as oleic acid, which target cancer cells (Svensson et al., 2000; Svanborg et al., 2003). These complexes are reminiscent of protein-SDS complexes since they involve a protein shell wrapped around a core of fatty acid arranged in a micellar form. Such a core-shell structure is formed by a large group of different proteins with oleic acid, and we have introduced the term *liprotide* for this class of complexes (Kaspersen et al., 2014). However, oleic acid does not readily form micelles at neutral pH, making it easier to study the stepwise unfolding and reconfiguration of aLA by using SDS instead.

Addition of SDS in low concentration to a solution of aLA results in a partially unfolded, molten globule state, whereas addition of SDS above the critical micelle concentration leads to partially structured protein-surfactant complexes (Halskau et al., 2005). Previous multidisciplinary studies of the stepwise unfolding of aLA showed two separate binding steps, in accordance with general protein behavior (Otzen et al., 2009). After initial binding of a few SDS molecules, the first main transition corresponds to exothermic binding of 4.4 SDS molecules, and is dominated by a secondary and tertiary structural denaturation of the protein and initiation of formation of SDS clusters or hemi-micelles bound to the protein. The second step corresponds to more modest tertiary protein unfolding and a reduced exothermic heat flow. In the middle of the second step, at the point of the lowest heat flow, 25 SDS molecules are bound per protein. According to isothermal titration calorimetry, a total of 43 SDS molecules bind to aLA. Up to the cmc, the log of the rate constant of unfolding of aLA increase linearly with the SDS concentration, but the slope is different in the first and second step, emphasizing that different structural changes are matched with different unfolding characteristics (Otzen et al., 2009). At saturation, 0.88 g of SDS are bound per gram of aLA which is common for proteins with disulfide bonds since the disulfide bonds restrict unfolding of the protein and thereby binding of SDS (Pitt-Rivers and Impiombato, 1968).

To elucidate how these changes at the level of the protein are accompanied by overall changes in the protein-SDS complex, we here combine synchrotron SAXS kinetics with Trp fluorescence and near-UV CD. SDS and protein were mixed in ratios corresponding to the end of the first step, the middle of the second step, the end of the second step, and at SDS saturation (Otzen et al., 2009). The results reveal an unfolding mechanism, which is broadly similar to that of bLG, suggesting a general mechanism of unfolding by SDS, irrespective of the sensitivity of the protein toward SDS.

## Experimental Methods

### Materials

Sodium dodecyl sulfate (SDS, ≥99.0%), bovine α-lactalbumin (aLA, >85%; single-band purity by SDS-PAGE indicates no significant protein contaminants) and all buffer components were from Sigma Aldrich (St. Louis, MO). All solutions were prepared in 20 mM NaHPO_4_ pH 7.0, 5 mM EDTA, leading to Ca^2+^-depleted aLA, i.e., apo-aLA. Concentrations of SDS used to obtain different ratios of SDS:aLA are provided in Table 1.

**Table 1:** Concentration of SDS used for different samples^a^.

**SDS per aL**	***c*_**SDS**_ (mM)**	***c*_**SDS, free**_ (mM)^**b**^**	**Binding number *N*^**b**^**	**Binding point^**b**^**
9	3.2	1.00	6.2	End of first step
27	9.5	1.45	22.9	Middle of second step
40	14.1	2.41	33.2	End of second step
60	21.2	2.9	52.2	Saturation^c^

c_SDS_ is the total SDS concentration and c_SDS, free_ is the concentration of free (not bound to protein) SDS molecules.

a c_aLA_ = 5.0 mg/mL (0.35 mM) for all samples. Buffer was 20 mM NaHPO_4_ pH 7.0, 5 mM EDTA.

b Based on ITC binding curves reported in (Otzen et al., 2009).

c *Corresponding to a complete return to baseline of the enthalpic signal; at 30 and 150 μM aLA, this occurs at 4.5 and 11 mM SDS, respectively*.

### Trp Fluorescence and Circular Dichroism

Full wavelength scans with far- and near-UV CD were performed on a Chirascan spectrometer (Applied Photophysics, Leatherhead, Surrey, UK) with a concentration of 1.0 mg/mL aLA and using a 0.02 and 1 cm quartz cuvette, respectively. Trp fluorescence was measured at 1 mg/mL aLA on a LS-55 Luminescence spectrometer (Perkin Elmer, UK) in a 1 cm quartz cuvette. Excitation was done at 280 nm using a scan speed of 200 nm/min. In all cases an average based on 3 scans is shown.

### Stopped-Flow Kinetics by Trp Fluorescence and Circular Dichroism

The kinetics of the protein unfolding after mixing of protein and surfactant solutions were measured on a Chirascan spectrometer with a stopped-flow mixing accessory (Applied Photophysics, Leatherhead, Surrey, UK) equipped with a Hg-Xe lamp. Trp fluorescence was followed with an excitation of 297 nm and a 2 nm bandwidth and emission measured using a 355 nm cut-off filter, while near-UV circular dichroism (CD) was followed at 297 nm with a 2 nm bandwidth. The pathlength was 2.0 mm for Trp fluorescence and 10.0 mm for near-UV CD. Solutions of aLA were mixed with SDS at a 1:1 volume ratio to a final aLA concentration of 5.0 mg/mL. Four different final concentrations of SDS, corresponding to distinct transitions in the binding of SDS to aLA (Table 1), were used to follow protein unfolding, all at 24°C. The shown data are averages of 10 measurements.

### Stopped-Flow Kinetics by Small-Angle X-ray Scattering

Data were collected on the ID02 TRUSAXS beamline at the European Synchrotron Radiation Facility (ESRF) in Grenoble, France, with fast read-out, low-noise CCD detector (FReLoN). Solutions of protein and surfactant were mixed at a 1:1 ratio, 200 μL of each, using an SFM-400 stopped-flow apparatus (Bio-Logic Science Instruments, France) (Panine et al., 2006). Sample concentrations after mixing are given in Table 1. Each data frame was collected with an X-ray exposure time of 5.0 ms, and with a lag time between frames for detector readout of 190 ms. The deadtime, which gives the shortest kinetic time accessible, was 2.5 ms. Kinetic times between 2.5 and 190 ms were covered by repeating the mixing and data collection while introducing a time delay between the time of mixing and collection of the first data frame. The sample-detector distance was 1.0 m, and the X-ray wavelength was λ = 1.00 Å, which results in a covered *q* range of 0.01–0.5 Å^−1^, where *q* is the momentum transfer of the scattered X-rays, defined as *q* = 4πsinθ/λ, and 2θ is the scattering angle. The 2D data were azimuthally averaged with the beamline software and converted to absolute scale using the scattering from pure water at 20°C. To improve statistics, the data were rebinned by home-writtem software to give data points that are approximately equidistant on a logarithmic *q* scale. Guinier fits were made to obtain model-independent information and further model-independent information was obtained by performing indirect Fourier transformation (Glatter, 1977; Pedersen et al., 1994) to obtain the pair distance distribution function *p*(*r*), which is a histogram of distances between pair of points, weighted by the excess electron density at the points.

### Modeling of SAXS Data

**Figure 1:**
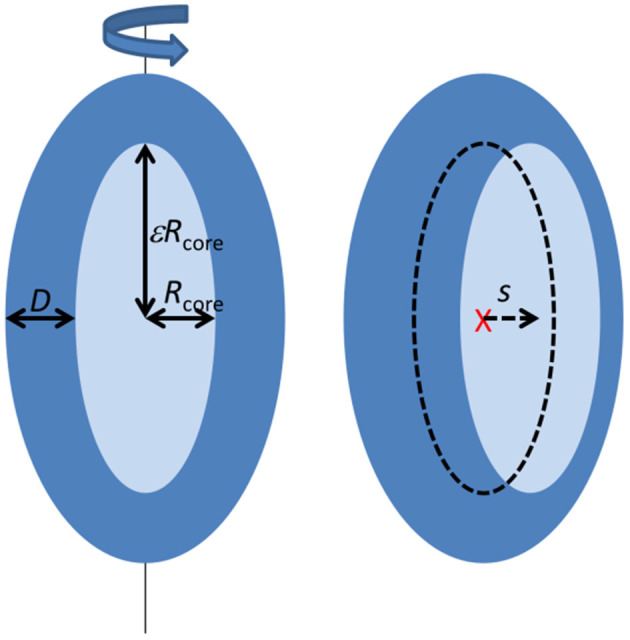
Illustration of the structural model applied to fit the SAXS data (see text).

Most of the scattering data at late stages show an oscillation characteristic for core-shell particles. They were analyzed using a model described in (Mortensen et al., 2017; Pedersen et al., 2019) (see Figure 1). The core-shell model particle (Figure 1) consists of a prolate ellipsoidal core of revolution with an equatorial radius *R*_core_ and axis ratio ε, (resulting in a polar radius of ε*R*_core_), and with a shell of thickness *D* along each of the three axes of the ellipsoid. For the SAXS data corresponding to the earliest times, the minimum before the oscillation is not very deep, suggesting a lower symmetry of the complex, which was introduced into the model by allowing a shift *s* (*s* < *D*) of the core center position relative to the center of the outer ellipsoid as defined by the shell. This then results in a shell, which is non-symmetrically distributed around the core. The interfaces between core and shell, and between shell and solvent were graded. In the model this was accounted for by multiplying the respective scattering amplitudes by Gaussians, exp(−*q*^2^σ^2^/2), where σ is the width of the interfaces. The mathematical expressions for the scattering form factor are given in (Mortensen et al., 2017; Pedersen et al., 2019). It should be noted, that the association of the protein with the micelle probably also involves some disorder and flexible polymer configurations, which may add to reduce the significance of the minimum. However, this was not included in the model as it cannot be distinguished from the asymmetric protein distribution and would add further fit parameters, which would be strongly correlated with the core displacement.

The data were modeled on absolute scale, so that the specific concentration and contrast of SDS tails, SDS head groups, and protein was taken into account [the details and values are given in Mortensen et al., 2017]. The core is assumed to consist of dry surfactant tails, and the shell consists of the surfactant head groups, protein, and water. The concentration of free SDS molecules, *c*_SDS, free_, is a function of both protein and SDS concentration. It was estimated from a previous study (Otzen et al., 2009) and the negligible scattering from these singly dissolved molecules was not included in the scattering model. The remaining SDS molecules and the protein was then associated with the core-shell complexes. In general, the free fitting parameters were then *R*_core_, ε, *D*, and *s*.

For conversion to absolute scale, the number of SDS molecules per complex, *N*_SDS_, was derived from the core volume and the specific volume of the SDS tail group, *V*_C12_ = 353 Å^3^ (Vass et al., 2003; Mortensen et al., 2017) using *N*_SDS_ = *V*_core_/*V*_C12_. The number of α-lactalbumin molecules per complex, *N*_α*L*_, was derived from the ratio between aLA and SDS in the complex, *N*_α*L*_ = *N*_SDS_ *c*_α*L*_/(*c*_SDS_ – *c*_SDS, free_), which assumes that the protein is evenly distributed in the complexes. The number concentration of complexes, *c*_complex_, was also derived from *N*_SDS_ as *c*_complex_ = (*c*_SDS_ – *c*_SDS, free_)*/N*_SDS_ and applied as a scale for the model scattering intensity, when it is converted to absolute scale. For some of the first frames, the protein per complex came out to be significant less than one, which is unreasonable. Therefore, the number concentration of complexes was set equal to the number concentration of proteins for these frames, thus fixing the number of protein per complex to one. The SDS was then evenly distributed on the complexes, which gives *N*_SDS_ and, consequently, also *V*_core_ as *V*_core_= *N*_SDS_ *V*_C12_. In practice, the core radius *R*_core_ was fitted and ε was calculated from the core volume. For the sample with an SDS:aLA ratio of 27, the data at early stages could only be fitted when only a fraction of the protein was in the complexes and the rest was as native protein. This was introduced in the model by fitting the concentration of protein in the complexes and adding the experimental scattering from native protein with a calculated scale factor, which ensured that the amount of protein was conserved. For the sample with an SDS:aLA ratio of 60, the protein per complex was fixed to one throughout the series.

The SAXS data had clear signs of concentration effects. Therefore, an effective hard-sphere structure factor (Kinning and Thomas, 1984), which depends on an effective interaction radius *R*_*HS*_ and an effective hard-sphere volume fraction η_*HS*_ were included in a decoupling approximation (Kotlarchyk and Chen, 1983). Some of the early frames showed signs of aggregation/dimerization and this was described by multiplying the intensity by the structure factor of a random flight chain (Burchard et al., 1970; Giehm et al., 2010; Rasmussen et al., 2019).

The consistency of the model was checked by calculating the amount of water in the shell, which also includes the SDS headgroups and the protein (see Results). For a physically reasonable model, there should be a relatively large fraction of water in the shell as the protein, and headgroups are not expected to pack densely. The protein and headgroups make up the ‘dry shell volume', *V*_shell, dry_, which was calculated from the aggregation numbers and the specific volumes of the protein, *V*_α*L*_, and of the SDS head group, *V*_head_, according to *V*_shell, dry_ = *N*_α*L*_*V*_α*L*_ + *N*_SDS_*V*_head_. The fraction of water in the shell was then derived from the total shell volume, *V*_shell_ = *V*_tot_ - *V*_core_ and the dry shell volume according to φ_wt, shell_ = (*V*_shell_ - *V*_shell, dry_)/*V*_shell_.

## Results

When SDS is titrated into aLA as monitored by ITC, two main binding transitions are observed at distinct SDS concentrations, followed finally by formation of a saturated SDS-aLA complex when 53 SDS molecules bind per protein (Otzen et al., 2009). Here we follow the kinetics of unfolding of aLA induced by mixing with a solution of SDS using stopped-flow SAXS, Trp fluorescence, and circular dichroism (CD). This is carried out by stopped-flow mixing at 1:1 vol/vol ratio. We follow the unfolding kinetics at four different SDS:aLA ratios (9, 27, 40, and 60). These ratios correspond to the end of the first step monitored by ITC, the middle of the second step, the end of the second step, and finally SDS saturation (Table 1). A more detailed description of the transitions between these different ratios and their structural interpretation can be found in our previous detailed study of the effect of SDS on the secondary and tertiary structure of aLA measured by Trp fluorescence, CD and stopped-flow (Otzen et al., 2009).

**Figure 2:**
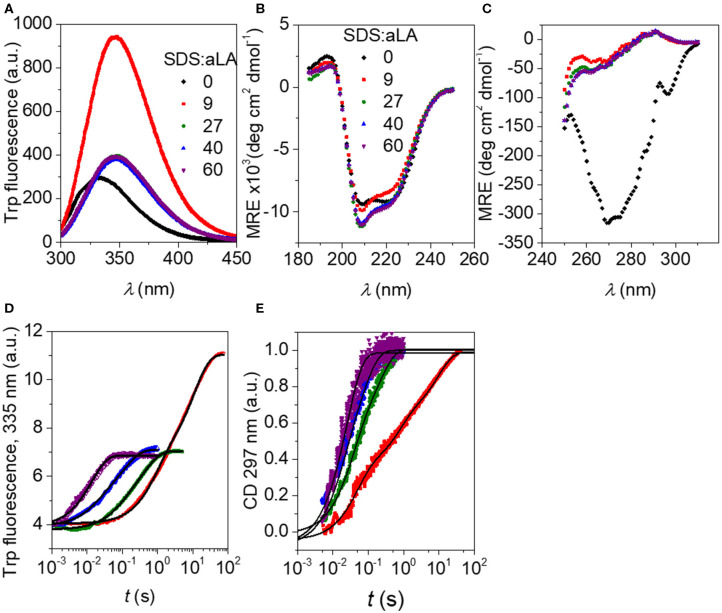
**(A)** Trp fluorescence vs. wavelength for SDS:aLA mixing ratios of 0, 9, 27, 40, and 60 (black, red, green, blue, and purple points). **(B)** Far-UV CD spectra for SDS:aLA mixing ratios of 0, 9, 27, 40, and 60 after equilibration. **(C)** Near-UV CD spectra for SDS:aLA mixing ratios of 0, 9, 27, 40, and 60 after equilibration. **(D,E)** Trp **(D)** and near-UV CD **(E)** time profiles for 1:1 stopped-flow mixing of aLA and SDS at 9–60 SDS:aLA mixing ratios. The lines are fits to double exponential functions as described in the text.

**Figure 3:**
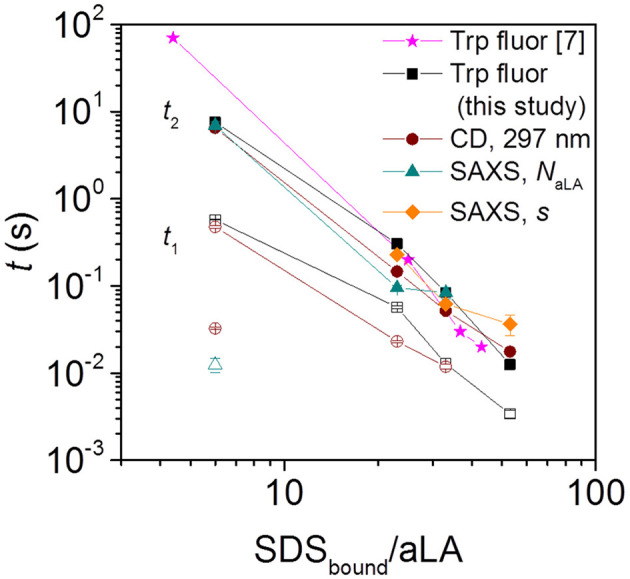
Half times *t*_1_ and *t*_2_ for aLA unfolding and formation of surfactant-protein complexes, observed by Trp fluorescence, near-UV CD, and SAXS. Filled and empty symbols of the same kind (e.g., square) represent slow and fast phases associated with that technique (for squares this is Trp fluorescence). The SAXS half times are obtained from the time evolution of parameters describing the number of aLA molecules per complex, *N*_aLA_, and the offset of the protein shell around the micelle core, *s*. Note that the stoichiometries refer to the compositions of the SDS:aLA complexes [based on ITC values Otzen et al., 2009] rather than bulk ratios. For the smallest SDS_total_:aLA ratio of 9 (which leads to a ratio of 6 SDS:aLA in the protein complex), CD (brown empty circle) and *N*_aLA_ values (cyan empty triangle) showed an additional fast exponential decay.

Trp fluorescence and CD traces are shown in Figure 2 together with the full spectra for the initial and final states. The four disulfide bonds restrict the extent to which aLA can become fully extended upon unfolding but both techniques show that the protein undergoes major structural changes upon addition of SDS. As observed previously (Otzen et al., 2009), these changes proceed more rapidly at higher SDS concentrations. The increase and red-shift in Trp fluorescence indicates a change in the local environment of the protein's four Trp residues. The red-shift is commonly seen when Trp residues become more solvent exposed. A large increase in intensity is seen at low SDS concentrations, similar to what is observed for other proteins such as bLG (Pedersen et al., 2019) and the cellulose-binding protein EXG:CBM (Højgaard et al., 2018). However, this increase is essentially canceled out at high SDS concentrations (Figure 2A). More information is provided by the almost complete disappearance of CD signal in the near-UV region upon addition of SDS (Figure 2C), indicating a loss in tertiary structure. The large changes seen in Trp fluorescence at low SDS concentrations indicate that the Trp environment in aLA is markedly different at low and high SDS concentrations. Our previous studies (Otzen et al., 2009) showed that the transition from 0 to 9 SDS:aLA was accompanied by a major increase in Trp fluorescence, complete loss of tertiary structure and a significant change in secondary structure, while the second step largely reflected a change in the polarity of the Trp residues and a completion of the change in secondary structure started in step 1. Satisfactory fits were obtained using double exponential fits to both the fluorescence and the CD data, indicating a multi-step process with characteristic half times *t*_1_ and *t*_2_. *t*_1_ and *t*_2_ for the two techniques are highly comparable and also close in value to previously reported values (Figure 3) (Otzen et al., 2009).

**Figure 4:**
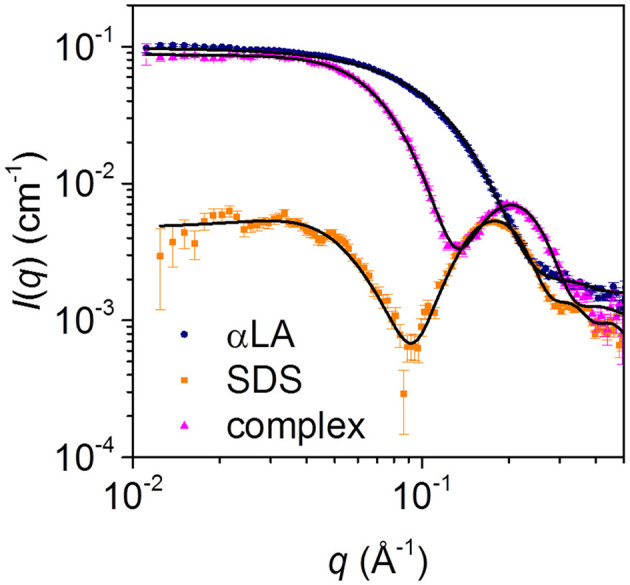
SAXS data for solutions of 28 mM SDS (orange squares), 0.7 mM aLA (blue circles) and a 1:1 mixture of the two (pink triangles), resulting in an SDS:aLA mixing ratio of 40.

Steady-state SAXS data for separate solutions of SDS micelles and free aLA, and for the final complex with mixing ratio SDS:aLA = 40, are shown in Figure 4. For SDS, the deep minimum in the data, together with the significant bump at *q* = 0.2 Å^−1^ agrees with published data and the expected globular micellar core-shell structure with a carbon-rich (and therefore electron-poor relative to water) core and a shell that is electron-rich (due to the sulfate groups and counter ions). For aLA, the data are in agreement with a homogeneous globular structure, and the data are fitted with a model of the known crystal structure (pdb code 1F6S). For the mixed surfactant-protein complex, the oscillation around *q* = 0.2 Å^−1^ again indicates the presence of core-shell structures in the solution, however, with a structure that is significantly altered compared to that of the SDS micelles.

**Figure 5:**
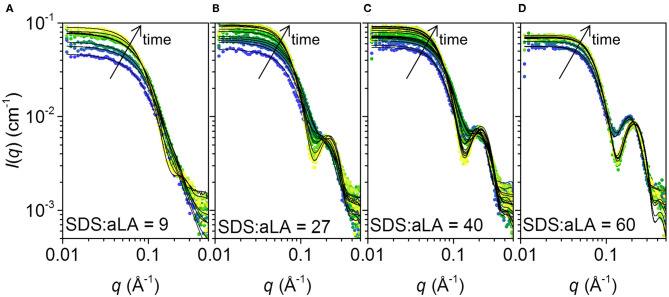
**(A–D)** Stopped-flow SAXS data plotted as scattering intensities vs. the momentum transfer *q*, collected after mixing SDS and aLA solution to obtain SDS:aLA ratios of 9, 27, 40, and 60, respectively. Time increases from blue to yellow. Selected data sets are shown. Corresponding staggered plots are shown in the SI (Figure S1) for better comparison of data and fits.

The SAXS data collected at different times after mixing at four different SDS:aLA ratios are shown in Figure 5. Staggered spectra at indicated time points are provided in the Supplementary Information Figure S1. At all SDS:aLA ratios, the scattering intensity for *q* → 0 (the forward scattering) increases with time, most pronounced in the beginning, indicating an average increase in mass of the protein-surfactant complexes. The rate of the increase, as well as the final value, increases with the SDS:aLA ratio. Except for the lowest SDS concentration, the late-stage scattering data agree qualitatively with the expected scattering from well-defined complexes with core-shell structure. Furthermore, the minimum in the scattering intensity becomes sharper with time, indicating formation of more symmetric protein-surfactant complexes. For the lowest SDS concentration, the SAXS data are more similar to that of a homogeneous globular structure, indicating that the SDS molecules do not form larger micelle-like structures and that the scattering is dominated by the protein.

To obtain model-independent information on the time development, the data were fitted by the Guinier expression $I\left(q\right)=I\left(0\right)\exp\left(-q^{2}R_{g}^{2}/3\right)$ to determine the forward scattering, *I*(*q*=0), and the radius of gyration *R*_*g*_. Plots of the values are shown in SI (Figure S2), and they display for *I*(*q* = 0) an increase with time in the beginning for *t* < 10 s for all samples. The sample with the lowest amount of SDS continues to increase throughout the time series. For the samples with SDS:aLA ratios 27 and 40, the values level off around the same value, whereas for SDS:aLA ratio 60, they level off at a somewhat lower value. It should be noted that the modeling (see below) reveals that there are structure factor effects, and that these change with time due to the contraction of the protein shell with time, so that this influences the values. The radius of gyration display a small tendency of a decrease with time, in agreement with a contraction of the shell. Additionally, the values at late stages are slightly decreasing with the amount of SDS, in agreement with an increased contribution from the hydrocarbon tails, which have a negative excess contrast. However, the structure factor effects and the inhomogeneous contrast are to some extent masking the structural changes. Further model-independent information was obtained by performing indirect Fourier transformation. A subset of the resulting pair distance distributions functions is shown in Figure S3. The analysis revealed presence of structure factor effects for the data with 27, 40, and 60 SDS per aLa and therefore the *q* range was limited (Glatter, 1979) to values larger than, respectively, 0.03, 0.04, and 0.05 Å^−1^. For 9 SDS per aLA, the functions show a gradual increase with time in agreement with the forward scattering determined in the Guinier analysis. The functions for larger amounts of SDS display all a shoulder or a minimum, reflecting the inhomogeneous structure of the complexes in agreement with a core of hydrocarbon chains surrounded by a shell of SDS headgroups and protein. Overall, also these functions increase with time in agreement with an increase of SDS aggregation number and/or number of aLA per complex with time.

For the further modeling and analysis, we first consider the simplest possible scenario, where there are no intermediate structures present. If the only species present are the SDS micelle, the native protein, and the complex structures, it should be possible to fit the data with a linear combination of scattering from SDS micelles, folded aLA and the final complexes. This approach, however, was not successful, as it resulted in poor fit quality for intermediate times, as well as incorrect values for the total aLA concentration (Figure S4). Instead, we adopted the core-shell model, based on a surfactant micelle structure decorated with protein on its surface. This model was developed in our recent study of unfolding and re-folding of β-lactoglobulin (Pedersen et al., 2019) and of SDS-aLA complexes (Mortensen et al., 2017). The shell consists of protein and surfactant head groups, and the core consists of the hydrophobic surfactant tail groups. To account for asymmetric distribution of the protein on the micelle, a fitting parameter was introduced that allows the core to be offset with respect to the center of the structure.

The model accounts specifically for the volume and scattering length density of each of the solution components, and can therefore be fitted to the data on an absolute scale, *i.e.*, using the actual concentrations of protein and SDS and their individual specific volumes to calculate the scattering contrast. For the early frames, the condition of a minimum of one protein per complex was imposed. The model fits are shown as solid lines together with the data in Figure 5. Overall, there is good agreement between data and model and the reduced chi-squared values are in the range 1–4 (Figure S5).

In order to get good fits at an SDS:aLA ratio of 27, a fraction of free protein had to be included, as already mentioned. At the lowest SDS:aLA ratio of 9, fewer features are observed in the scattering patterns at early times. This makes it possible also to fit a much simpler model, describing homogeneous ellipsoidal particles, to the data with only moderate loss of fit quality. Free protein is likely present at the early times, given its presence at an SDS:aLA ratio of 27. However, the featureless scattering data meant that we could not detect it. At the latest times, however, somewhat sharper features appear in the scattering pattern, and the symmetric core-shell structure is the only relevant model. As SDS is bound to the protein at all times, the results for the core-shell model are given for the sample with SDS:aLA ratio of 9, although one should be cautious about over-interpreting the results from the model for this sample.

**Figure 6:**
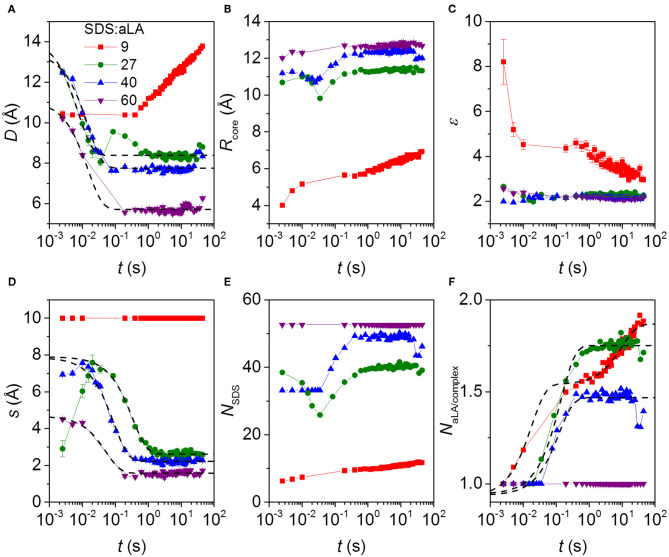
Parameters from model fits to kinetic SAXS data after stopped-flow mixing of aLA and SDS in mixing ratios SDS:aLA 9 (red points), 27 (green points), 40 (blue points), and 60 (purple points). **(A)** The shell thickness, *D*. **(B)** The core radius *R*_core_. **(C)** Axis ratio of ellipsoidal core, ε. **(D)** Shift *s* of the core relative to the center of the outer ellipsoid in the core-shell model (see text). **(E)** Number of SDS molecules per micelle in protein-surfactant complex, *N*_SDS_. **(F)** Number of aLA molecules per complex, *N*_α*LA*_.

The fitting parameters and derived parameters obtained from the model fits are shown in Figure 6. The results reveal a rather complex dependence of the parameter on the SDS:aLA ratio. For the structure of the final complexes, the number of SDS molecules per complex increases with the SDS:aLA ratio, with the cores radius increasing and the aspect ratio slightly decreasing. The opposite trend is seen for the number of aLA molecules per complex, *N*_α*L*_, which decreases with the SDS:aLA ratio. The values of *N*_α*L*_ range between 1 and 2. Non-integer values do not imply that only part of a protein is attached to an SDS micelle, but rather reflect an average composition of the complexes. The behavior with the number of proteins per complex decreasing with increasing amounts of SDS probably reflects the drive to attain an optimal number of SDS molecules in the central micelle. As a result, more than one aLA has to be attached to each micelle for SDS:aLA ratios of 27 and 40, whereas it fits exactly with one protein at saturation at a ratio at 60.

The time dependence is quite complex. Overall the thickness of the shell, *D*, and the core shift, *s*, decrease as a function of time, except for an SDS:aLA ratio of 9, where *D* increases first slowly and then faster (while *s* is kept constant, due to the lack of prominent core-shell features in the data at this low SDS:aLA ratio) due to an increase in the number of proteins per complex. Note that an exception to the general decrease of *s* is that the core shift increases slightly during the first 2–3 time frames at 27 and 40 SDS:aLA. We attribute this slight increase to the existence of free aLA at these very early stages of the reaction, which will artificially skew the average shape of the complex. The overall decrease of *D* and *s* with time can be explained by a very disordered asymmetric protein distribution in the beginning, which gradually contracts and develops into a more symmetric shell. This asymmetry potentially affects the model in a way which *s* becomes more uncertain. In general, the core radius increases slightly with time and the axis ratio of the core decreases slightly with time. The combined effect of these results in an increase of the SDS aggregation number with time (not observed for an SDS:aLA ratio of 60, where the aggregation number is fixed by the condition of having exactly one protein per complex). It should be noted that interface smearings were included in the model. For an SDS:aLA ratio of 9, the σ values were fixed at 1.0 Å for the core-shell interface and 2.0 Å for the shell-solvent interface. For SDS:aLA ratios of 27 and 40, the σ values were the same for the initial frames, and when the fit quality became worse, they were increased to 2.0 and 3.5 Å, respectively. We interpret this to represent “buckling” of the protein as it contracts within the shell. The data for the sample with a ratio of 60 was fitted with the latter values throughout the series.

The time dependence of some additional parameters are displayed in Figure S6. The number of micelle per complex was for an SDS:aLA ratio of 27 and 40 equal to 2.0 in the first frame and then decreases rapidly to 1.0. This was for the micelle separation fixed at 66 Å. For the two other ratios, the number of micelles per complex was 1.0 throughout the series. The effective hard-sphere volume fraction is also displayed in Figure S6. For an SDS:aLA ratio of 9, it is relatively constant, whereas it decreases strongly initially for the other ratios. This decrease follows the decrease of the protein shell width at early stages and the smaller volume of the complexes associated with this. The effective hard-sphere radius was fixed at 43 Å for SDS:aLA ratio of 9, 27, and 40, and at 30 Å for a ratio of 60. For the sample with an SDS:aLA ratio of 27, not all protein was initially in the complexes (as mentioned earlier). The development of the concentration of protein in complexes for this sample is also shown in Figure S6. It starts out at 3 mg/mL and changes rapidly so that all protein (5.0 mg/mL) is in the complexes.

The Trp fluorescence and CD data both provide two half times *t*_1_ and *t*_2_, each of which decreases with increasing SDS:aLA ratio. This indicates two kinetic steps in complex formation. The SAXS data show that protein-surfactant complexes are formed already within the deadtime of the stopped-flow experiments. That is, the fastest process related to *t*_1_ is not captured by the SAXS data, but it is likely associated with the earliest binding step, where individual SDS molecules bind to aLA. Structural changes associated with the second step can be elucidated by examining the temporal development of the structural parameters derived from modeling of the SAXS data. The early time developments show that the core displacement decreases and the number of protein molecules per complex increases. The *s* values show that initially, the surfactant-protein complexes are rather asymmetric, signifying that the protein is mainly adsorbed to one side of the SDS micelle. The values of *s* then decreases, indicating that the protein is rearranged around the micelle, resulting in more symmetric complexes. The half times related to *s* are generally comparable to *t*_2_ of the unfolding. This shows that the protein rearrangement is associated with the second unfolding step (Figure 3). The half times related to the number of proteins per complex is similar to that for the core displacement, which shows that an increase in the size of complexes is taking place at the same time as the protein rearrange into a more symmetric distribution.

There are also some faster changes observed for some of the parameters derived from SAXS. The shell thickness, *D*, and the number of micelles per complex decreases in the first frames in a very similar and nearly concentration independent manner. Fitting the decay in shell thickness *D* gives half times of (5.1 ± 1.4) ms, (10 ± 1) ms, and (10 ± 1) ms for 27, 40, and 60 SDS/aLA, respectively. Thus, there is no apparent systematic dependence of SDS:aLA ratio, so the process cannot directly be related to the *t*_1_ process as probed by Trp fluorescence and CD, but might simply be structural rearrangements that occur concomitant with the changes in secondary and tertiary structure.

## Discussion

### A Two-Step Model for Unfolding of aLA by SDS: Redistribution of Proteins and Growth of Complex Size

**Figure 7:**
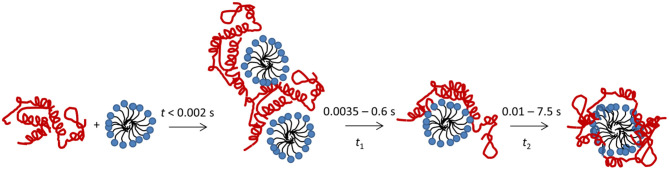
Schematic representation of the unfolding process after mixing of aLA (red) and SDS (blue).

Here we describe the combined use of three different stopped-flow techniques (SAXS, CD and Trp fluorescence) to monitor the SDS-unfolding of aLA. This approach provides a very comprehensive overview of the structural changes associated with this transition at the level of both protein and surfactant. We summarize our results for the process of complex formation and protein unfolding in Figure 7. Complexes with a distinct core-shell structure are formed already within the 2.5 ms deadtime of the kinetic experiments. The SAXS data show that these initial complexes are highly asymmetric as indicated by the offset core, *i.e.*, the protein is attached mostly to one side of the micelle. A low degree of clustering is observed, and the structural model describes dimers of SDS-aLA complexes. Within the first 10 ms, however, the clusters dissociate and the shell thickness of the complexes decreases, indicating tighter association of the protein with the micelle surface. This dissociation of clusters and decrease in shell thickness is not visible for the lowest SDS:aLA ratio, but under these circumstances, the core-shell structure is less prominent, making it difficult to make robust structural conclusions relating to the fine structure of the complex. We have previously carried out steady-state SAXS measurement on aLA-SDS complexes at concentrations close to the aLA:OA 1:27 and 1:60 ratios (Mortensen et al., 2017). Here, *D* decreased from 12 to 7.5 Å, ε decreased from 4.4 to 1.9 and *R*_core_ increased from 10.8 to14.3 Å in good agreement with the steady-state values presented here. Overall, the SAXS analysis of the final complexes highlights a quite thin shell and a high symmetry, reflected by the deep minima in the scattering curve. This suggests that despite the constraints provided by the four disulfide bonds, the protein is homogeneously distributed on the surface of the SDS micelle within the resolution provided by SAXS.

The protein unfolding, as followed by Trp fluorescence and CD, takes place in two consecutive steps, associated with half times *t*_1_ and *t*_2_. The second of these correspond to further structural rearrangement of the complex, resulting in larger complexes with higher numbers of both SDS and aLA molecules, and with a more symmetric shape, indicating an even distribution of protein on the micelle surface. The two observed steps in the unfolding process both occur at all the tested SDS concentrations.

### Comparison With bLG: aLA Undergoes Faster Unfolding and Forms More Proteins per Complex but Follows the Same Overall Process

Our results provide an opportunity to compare with similar studies recently performed on bLG (Pedersen et al., 2019). For both proteins, the speed of the unfolding process increases with increasing SDS:protein ratio due to the ability of SDS to associate more rapidly into higher-order structures at higher concentrations. However, the entire unfolding process is significantly faster for aLA (half-times of 0.02–0.15 s at SDS:aLA ratios of 23–52, according to near-UV CD measurements) than the unfolding of bLG (half-lives of 0.36–1.86 s at SDS:bLG ratios of 22–63), in accordance with previous spectroscopic studies and the high overall susceptibility of aLA to unfolding by SDS and other surfactants (Otzen et al., 2009). This reflects the underlying difference in structure between the two proteins; bLG is held together by global interactions between β-strands, which are not stable as individual isolated strands in SDS micelles, unlike the α-helices making up most of the aLA structure, which are stabilized by local H-bonds and therefore transfer more favorably to the apolar part of a micellar environment.

For bLG the proteins and micelles initially cluster together, followed by dissociation into asymmetric core-shell structures. This is also seen at the intermediate SDS:aLA ratios of 27:1 and 40:1 but not at the lowest (9:1) and highest (60:1) ratios. aLA (net charge −6) and bLG (net charge −8) have a similar net charge under these conditions. Thus, the extent of aggregation does not seem to be related to electrostatic interactions but may reflect the size and structure of the protein. bLG is 30% larger than aLA in terms of residues, thus bLG could therefore simply bind more micelles than aLA. Alternatively, the β-sheet rich protein bLG may be more prone to forming intermolecular contacts via β-strand H-bonding than the much more helical protein aLA. Also, given that bLG clustering and dissociation occurs within 0.03–0.1 s and the process occurs much faster for aLA, it is most likely that these processes occur so quickly at the SDS:aLA ratio of 60 that they escape detection.

For unfolding of bLG with SDS, a third and much slower process was observed by fluorescence and CD but not with SAXS. This must reflect changes to bLG at the level of secondary and tertiary structure, which do not lead to significant changes in the protein-surfactant complex. Accordingly, we have attributed this process to minor rearrangements of the protein on the micelle surface. This third step was not observed for aLA and could be explained by aLA being an almost purely helical protein. For aLA, such a “ready-made” secondary structure eliminates the need for structural transition from β-sheet to α-helix, which bLG has to undergo to ensure optimal micelle binding.

In addition to the initial micelle clustering which could be included in the model for SDS:aLA ratios of 27:1 and 40:1, a large fraction of the complexes contain two aLA molecules at the end of the measured time window, except for the highest SDS:aLA ratio of 60. This contrasts with bLG which at all SDS concentrations ended up with one protein per complex. It is likely that the SDS micelle simply cannot accommodate more than one bLG protein because of its larger size compared to aLA. At SDS saturation, both aLA and bLG, however, bind only one protein per micelle because of the excess of SDS.

### Parallels to Liprotides: Similar Core-Shell Structure but no Free OA Micelles at Neutral pH

The liprotide complexes that are formed between aLA and oleic acid (OA) have a similar core-shell structure to the structures formed between aLA and SDS (Kaspersen et al., 2014). The protein binding to OA micelles in the liprotides is however somewhat more flexible with part of the protein being in a more flexible conformation. Nevertheless, the secondary and tertiary structural changes seen for SDS- and OA-induced unfolding are very similar, suggesting underlying structural similarities. Therefore, we predict that the rapid rearrangements seen for SDS induced unfolding of aLA will be observed for OA. Nevertheless, this is not experimentally very tractable, since OA does not spontaneously form micelles at neutral pH. Although an increase to pH 11 favors micelle formation, other structures are also favored under these conditions, which obscures SAXS interpretation. Following the structural changes with far-UV CD and Trp fluorescence should, however, be possible, even if OA is present in several aggregated structures.

## Data Availability Statement

All datasets generated for this study are included in the article/Supplementary Material.

## Author Contributions

GJ, JNP, DO, and JSP conceived and designed the experiments, analyzed the data, and wrote and edited the manuscript. GJ and JNP performed the experiments and performed computational analysis. All authors contributed to the article and approved the submitted version.

## Conflict of Interest

The authors declare that the research was conducted in the absence of any commercial or financial relationships that could be construed as a potential conflict of interest.

## Abbreviations

aLA: α-lactalbumin
CD: circular dichroism
ITC: isothermal titration calorimetry
SAXS: small-angle X-ray Scattering.
