# Supplementary material for: Multi-Step Unfolding and Rearrangement of α-Lactalbumin by SDS Revealed by Stopped-Flow SAXS
Source: Front Mol Biosci. 2020 Jul 10;7:125. doi: 10.3389/fmolb.2020.00125 (PMC7366515; doi:10.3389/fmolb.2020.00125)
Supplement: Supplementary file 1 [file Data_Sheet_1.DOCX]

**Supplementary information**

**Multi-step unfolding and rearrangement of α-lactalbumin by SDS revealed by time-resolved SAXS**

Grethe Vestergaard Jensen, Jannik Nedergaard Pedersen, Daniel Otzen, Jan Skov Pedersen


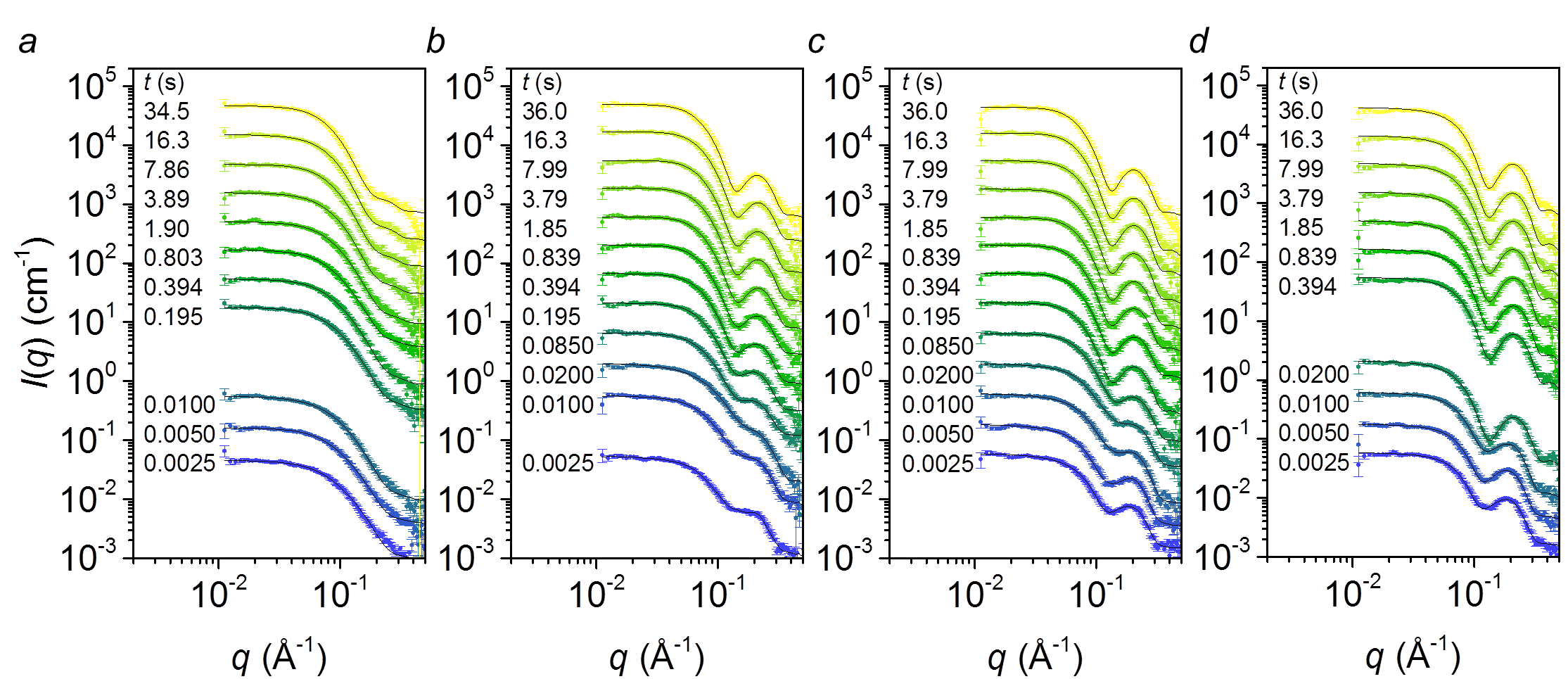


Fig. S1. Staggered plots of the SAXS data for selected sets. The bottom plots are on absolute scale, and the consecutive plots are multiplied by factors of 3. The times are given in seconds. From left to right, SDS:aLA ratios of 9, 27, 40, and 60,respectively. The lines are fits by the model as described in the text.


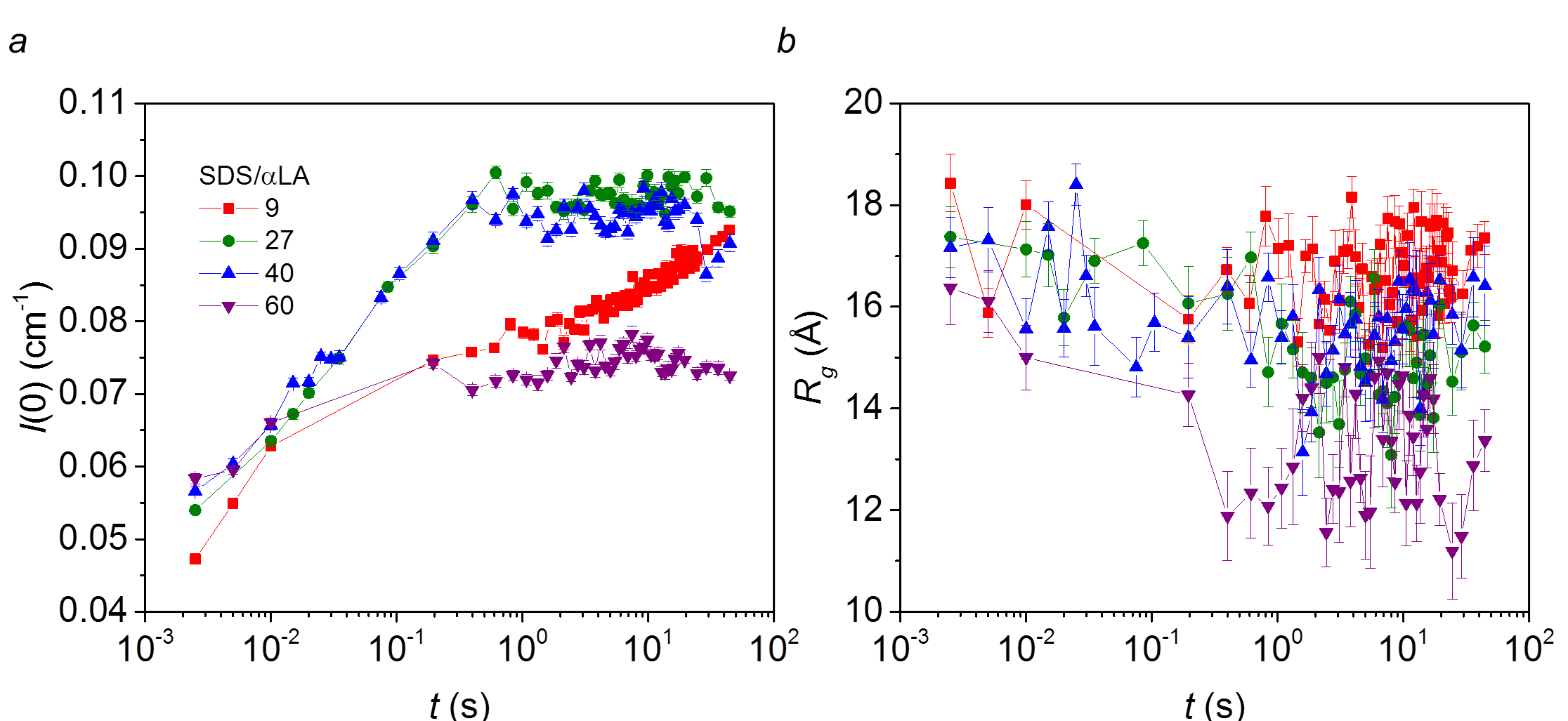


Figure S2: Result from Guinier fits for the forward scattering and radius of gyration.


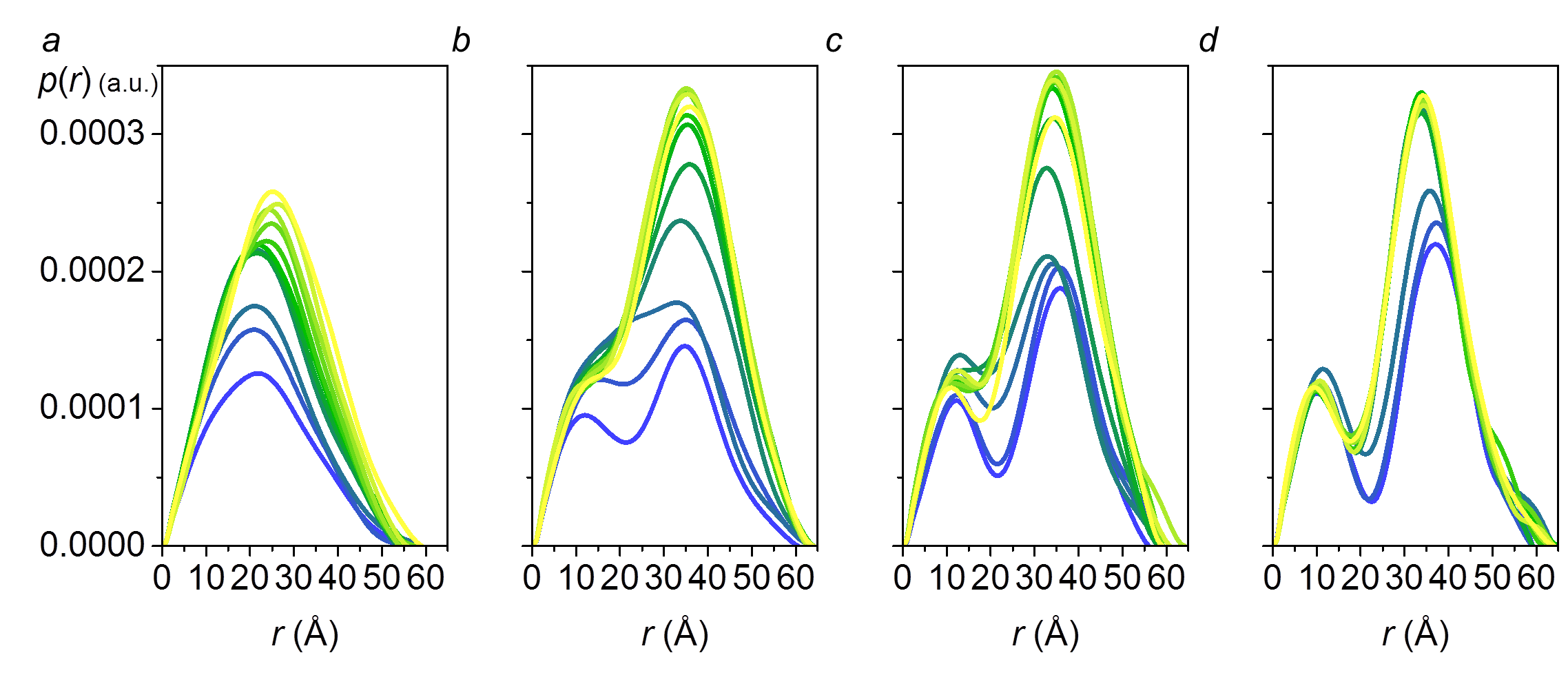


Fig S3: Distance distribution functions for the data sets in Fig. 5 and S1. From left to right, SDS:aLA ratios of 9, 27, 40, and 60,respectively. Time increases from blue to yellow.

**Linear combination fit:**

An attempt to fit with a linear combination of SDS micelles, native aLA, and final complexes was not successful (Fig. S6): For 27 and 40 SDS per aLA, at intermediate times a decrease in fit quality is observed. During this time, the linear combination suggests a peak in concentration of free aLA. Initially the total fraction of aLA content (complex + aLA) is considerably less than unity, for all SDS concentrations.


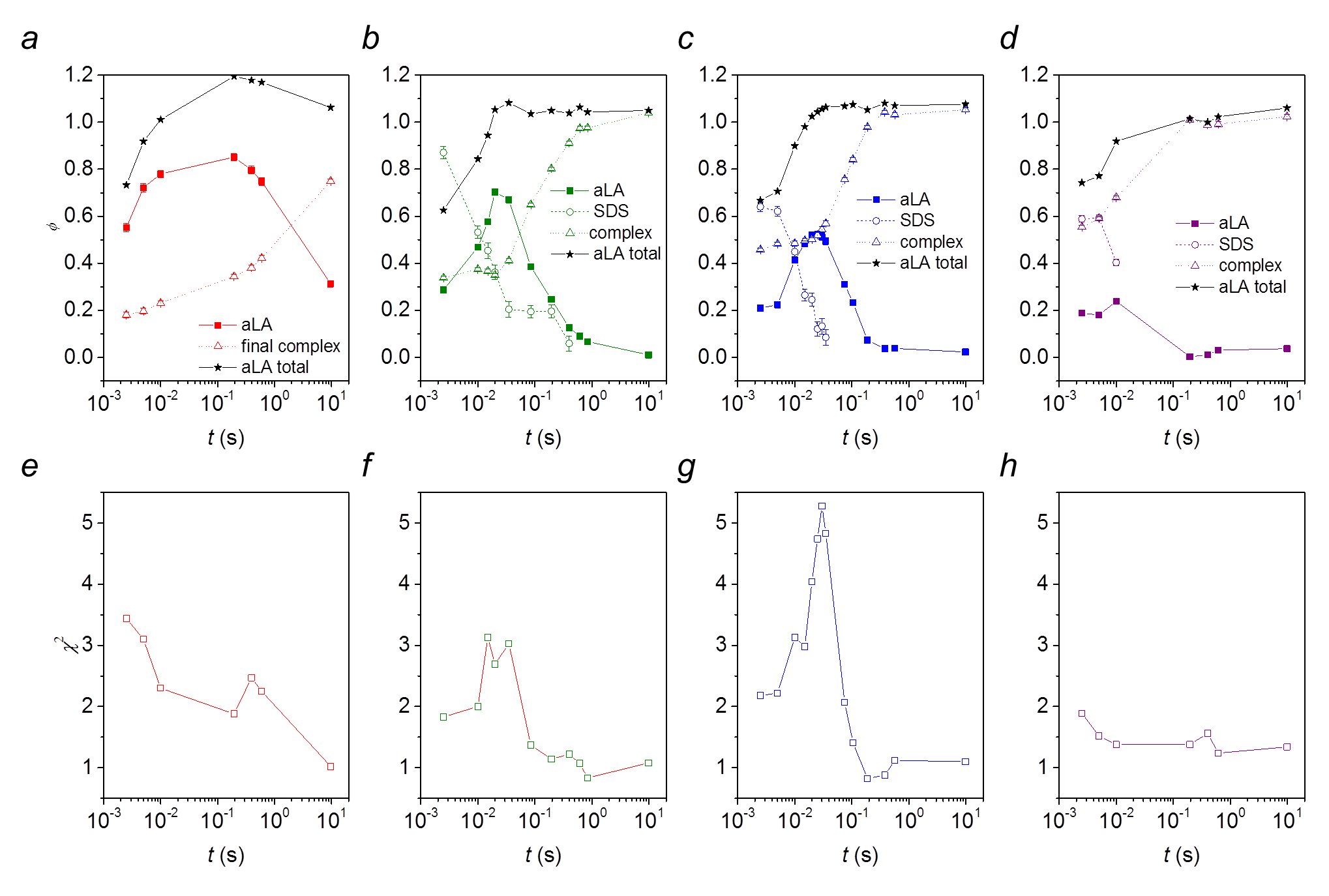


Fig. S4: Contributions from aLA, SDS, and the final complexes respectively for the linear combination fits, as well as the total contribution from aLA given by the sum of aLA and complexes. See main text for details. *a-d:* From left to right, SDS:aLA ratios of 9, 27, 40, and 60,respectively. *e-h*: Corresponding chi-squared values for the fits.


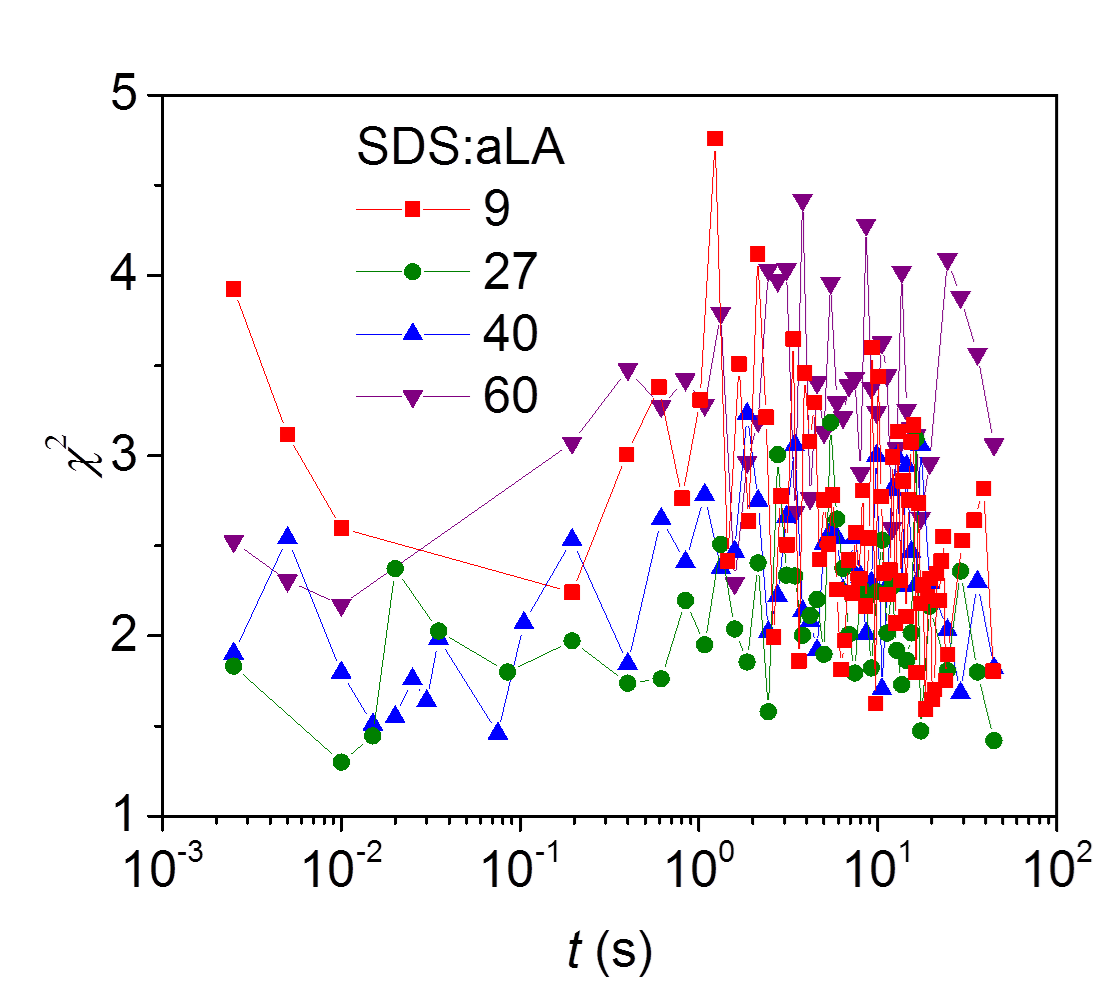


Figure S5: Chi-squared values for the model fits, using a core-shell model, based on a surfactant micelle structure decorated with protein on its surface. The model was fitted to the data on an absolute scale. See main text for details.


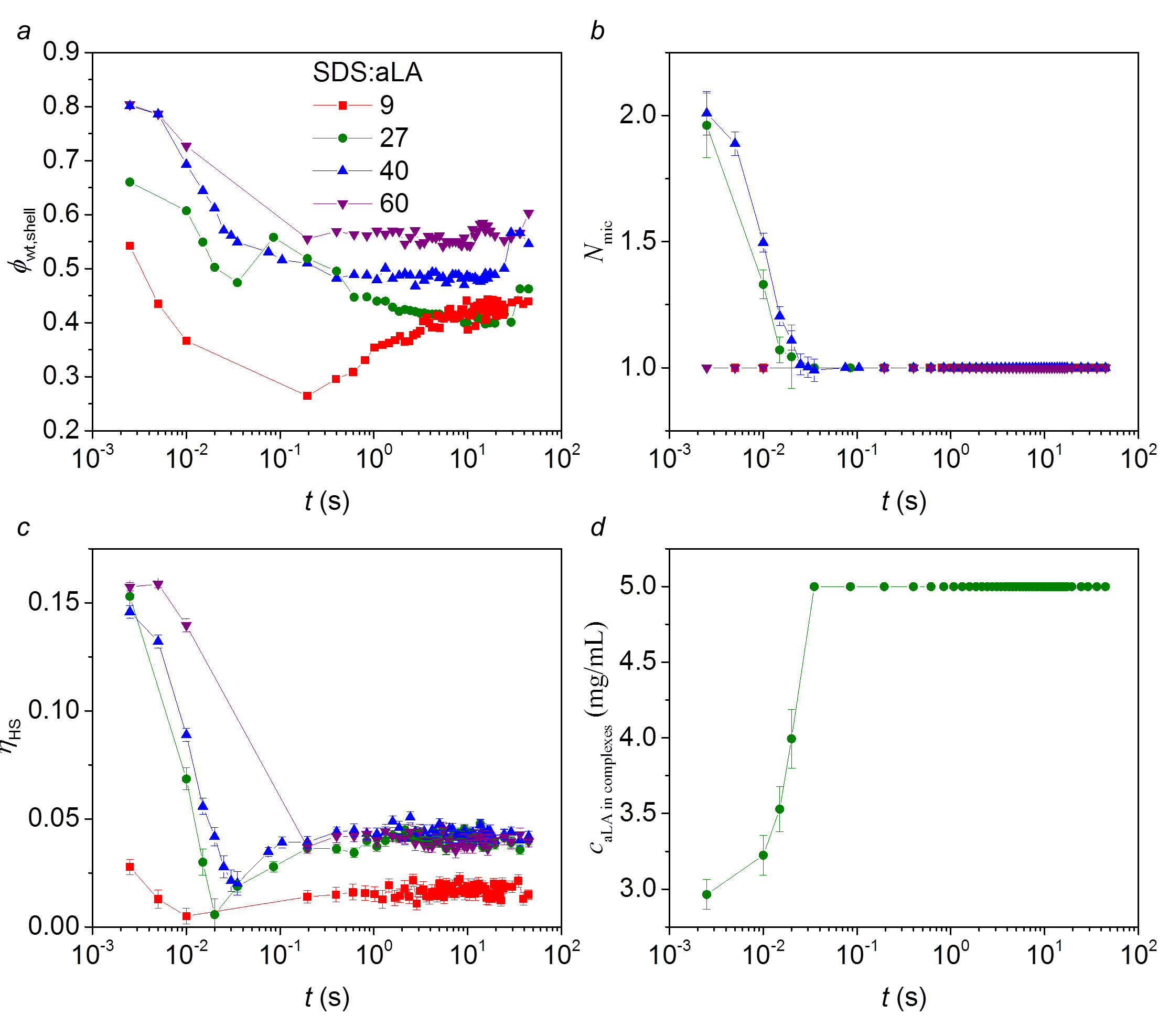


Figure S6: Time-dependent changes in parameters resulting from model fits to the kinetic data. See main text for details. *a,* fraction of water in the complex shell, *φ*_wt,shell_. *b*, number of decorated micelle complexes per cluster *N*_mic_. *c*, effective hard-sphere volume fraction, *η*_HS_. *d*, concentration of aLA bound in complexes *c*_aLA in complexes_ (only fitted for 27 SDS/aLA, for all other ratios, all aLA was considered bound in complexes at all times).
